# Assessment and Documentation of Social Determinants of Health Among Health Care Providers: Qualitative Study

**DOI:** 10.2196/47461

**Published:** 2023-07-03

**Authors:** Brooks Yelton, Jancham Rachel Rumthao, Mayank Sakhuja, Mark M Macauda, Lorie Donelle, Michelle A Arent, Xueying Yang, Xiaoming Li, Samuel Noblet, Daniela B Friedman

**Affiliations:** 1 Department of Health Promotion, Education, and Behavior Arnold School of Public Health University of South Carolina Columbia, SC United States; 2 Center for Applied Research and Evaluation Arnold School of Public Health University of South Carolina Columbia, SC United States; 3 College of Nursing University of South Carolina Columbia, SC United States; 4 SC SmartState Center for Healthcare Quality Department of Health Promotion, Education, and Behavior Arnold School of Public Health, University of South Carolina Columbia, SC United States

**Keywords:** social determinants of health, SDOH, health equity, Healthy People 2030, interviews, thematic analysis, health care worker, health care provider, health equity, barrier, facilitator, qualitative study, web-based, patient health, well-being, community health status

## Abstract

**Background:**

Research clearly demonstrates social determinants of health (SDOH) impact health outcomes. Provider consideration of patient SDOH in prevention and treatment planning is critical for improved health care quality and health equity. Despite awareness of the connections between SDOH and improved population health, research demonstrates few providers document patient SDOH.

**Objective:**

This qualitative study aimed to better understand the barriers and facilitators of SDOH assessment, documentation, and referral in different health care settings and roles.

**Methods:**

Individual semistructured interviews were conducted with practicing health care providers in South Carolina between August 25, 2022, and September 2, 2022. Participants were recruited via community partners’ web-based newsletters or listservs using a purposive sampling design. An interview guide with 19 questions was used to explore the following research question: How do SDOH impact patient health and what are the facilitators and barriers experienced by multidisciplinary health care providers assessing and documenting patient SDOH?

**Results:**

Participants (N=5) included a neonatal intensive care unit registered nurse, a nurse practitioner, a certified nurse midwife, a family and preventive medicine physician, and a counselor (licensed clinical social worker) with careers spanning 12 to 32 years. Participant responses are presented according to the following 5 themes: participants’ understanding of SDOH for the patient population, assessment and documentation practices, referrals to other providers and community-based resources, barriers and facilitators of SDOH assessment and documentation, and SDOH assessment and documentation training preferences. Overall, participants were aware of the importance of including patient SDOH in assessment and intervention but noted a variety of institutional and interpersonal barriers to assessment and documentation, including time constraints, perceptions of stigma around discussion of SDOH, and limited referral protocols.

**Conclusions:**

Incentivizing inclusion of patient SDOH in health care must be facilitated from the top down, so assessment and documentation can be universally implemented in a pragmatic way that works for providers in a variety of roles and settings for the betterment of health care quality, health equity, and improved population health outcomes. Partnering with community organizations can serve to augment health care organizations’ resource and referral availability for addressing patients’ social needs.

## Introduction

Social determinants of health (SDOH) are defined by the United States’ Healthy People 2030 (HP 2030) as “the conditions in the environments where people are born, live, learn, work, play, worship, and age that affect a wide range of health, functioning, and quality-of-life outcomes and risks.” HP 2030’s data-driven national objectives categorize SDOH into 5 fundamental domains: economic stability, education access and quality, health care and quality, neighborhood and built environment, and social and community context [1]. Increased research interest in SDOH among the public health and medical community is driven by evidence SDOH clearly impact health outcomes. Between 30% and 55% of health outcomes are ascribed to SDOH [2].

Population health has become an increasingly critical focus of health care delivery, especially during the COVID-19 pandemic [3]. SDOH factors allow clinicians to consider potential contributors to poor health outcomes, reduce health disparities, and transform health care delivery through partnerships with community-based resources [4]. Using nonbillable diagnostic “Z codes”—specific to SDOH—provided by International Classification of Diseases, 10th Revision, Clinical Modification in electronic medical record (EMR) systems provides clinicians and their medical teams with the opportunity to identify and document SDOH issues, establish appropriate intervention plans, reduce costs, and enhance health care delivery with an eye toward improving health equity [5,6]. To improve health care quality and quality of life, structural health perspectives that recognize and engage with the experiences and backgrounds of diverse individuals are critical in closing equity gaps and reducing health disparities [7,8].

An empirical study of clinical application of Z codes in South Carolina before and during the COVID-19 pandemic found Z codes were only documented for 1.23% of patients in a large statewide sample [9]. This research demonstrated an overwhelming lack of attention to the social context of (ill) health. This was especially true for individuals requiring outpatient care relative to individuals who were hospitalized. Although EMR systems are an efficient method for clinical data collection, the uncertainty in nonclinical diagnostic Z code use and potential differences in assessment tools and data collection methods limit SDOH information quality [10]. Furthermore, time constraints associated with the medical care model, varied provider conceptualization of SDOH, institutional practices and priorities, and inconsistent knowledge or availability of referral resources inhibit universal assessment and documentation practice. A recent study found higher awareness regarding the need for documenting SDOH among providers in community health centers, social care–associated payment models, and those with greater knowledge of advanced functions within EMR systems [11]. Health service organizations have attempted to promote clinician documentation and implementation of SDOH diagnostic codes by providing improved SDOH standardized terminology and data principles for which Z codes are categorized [12]. However, data regarding the effectiveness and barriers of these efforts are limited.

This qualitative study aimed to examine health care providers’ experiences with assessment and documentation of the SDOH impacting patient health and well-being. Through in-depth interviews, our goal was to better understand the barriers and facilitators of SDOH assessment, documentation, and referral in different health care settings and roles.

## Methods

### Study Design

Qualitative research methods were used to address the following research question: How do SDOH impact patient health and what are the facilitators and barriers experienced by multidisciplinary health care providers assessing and documenting patient SDOH?

In total, 7 team members (BY, JRR, DBF, MAA, MMM, MS, and SN) and 2 clinical partners assisted in the development of a recruitment plan to leverage clinical-academic-community partner web-based newsletters or listservs in a purposive sampling design [13] deployed between July 12, 2022, and August 30, 2022; the opportunity was also advertised on partners’ social media (ie, Twitter). A snowball sampling strategy was also used [13]. Direct email invitations were sent to contacts identified by partners. Participants were eligible if they were active, English-speaking primary or behavioral care providers in South Carolina with direct patient involvement. Twelve interested participants reached out via email; 7 did not meet the criteria (5 served in administrative or management roles with no direct patient involvement, and 2 were not providers), while 5 participants met eligibility criteria and agreed to participate. There was no previous relationship between the authors and the participants. Participants were made aware of the authors’ credentials and the purpose of this research in the recruitment language.

An interview guide with 19 questions was iteratively developed by 7 team members (BY, JRR, DBF, MAA, MMM, MS, and SN) and reviewed by 2 partners to explore participants’ current knowledge about SDOH and use of International Classification of Diseases, 10th Revision, Clinical Modification Z SDOH codes. The interview guide was piloted with 2 providers, including a clinical partner (Multimedia Appendix 1).

Individual semistructured interviews lasting between 30 and 45 minutes were conducted between August 25, 2022, and September 2, 2022, by 1 study team member (BY) via Zoom; 1 team member (JRR) sat in on 1 interview to take notes. Interviews were recorded and transcribed verbatim through Zoom transcription services and imported into NVivo (version 12, 2022-2023; QSR International Pty Ltd) for analysis.

For codebook development, 1 team member (BY) initially matched concepts to the interview guide using a deductive approach. Two team members (BY and MMM) then independently reviewed and coded 3 transcripts each for comparison and finalization using an inductive approach. Intercoder agreement resulted from iterative discussions between the 2 coders. One team member (BY) then iteratively coded all 5 transcripts using semantic thematic analysis [14,15], and emergent codes were added to the codebook until no new codes were found [16]. Participant responses are presented according to the following 5 themes: participants’ understanding of SDOH for the patient population, assessment and documentation practices, referrals to other providers and community-based resources, barriers and facilitators of SDOH assessment and documentation, and SDOH assessment and documentation training preferences.

### Ethics Approval

This study was approved by the University of South Carolina Institutional Review Board (Pro00122161). Recruitment language providing a description of this study’s purpose, eligibility, procedure, and incentives for informed consent was disseminated through statewide partner e-listservs. Transcribed interviews were deidentified (participant 1, participant 2, etc), revised for accuracy, and imported into NVivo for data management by 1 team member (BY). Original interview audio files were securely stored in a password-protected, cloud-based storage application with limited team member access. Participants were provided a US $25 incentive for their time.

## Results

### Sample Characteristics

Participants (N=5) included a neonatal intensive care unit registered nurse, a nurse practitioner, a certified nurse midwife, a family and preventive medicine physician, and a counselor (licensed clinical social worker). Participants’ careers in health care ranged from 12 to 32 years, with 1 to 25 years of experience in their current roles. Three participants currently work within a large health care system, 1 participant works for a university health care system, and 1 participant works at a rural, 2-physician practice. Two participants also serve as faculty members involved in training the health care workforce within a university system (Table 1).

**Table 1:** Sample characteristics.

	Current role	Time in current role (years)	Length of career (years)
Participant 1	Staff nurse (health care system) neonatal intensive care unit	5	12
Participant 2	Nurse practitioner (health care system)	16	32
Participant 3	Certified nurse midwife (rural clinic)	25	30
Participant 4	Family and preventive medicine physician (health care system)	9	17
Participant 5	Counselor (health care system)	1	30

### Participants’ Understanding of SDOH for the Patient Population

Participants seemed to have a good understanding of SDOH and were able to reflect on how SDOH might impact their specific patient population. All participants reported SDOH are important to assess for improved treatment planning but described a range of assessment and documentation practices. Participants identified a variety of SDOH impacting their patients including general access barriers (time, transportation, and childcare), health care access and quality (affordability and provider’s time), education and health literacy, poverty and material constraints, food insecurity, and social connectedness. For example, 2 participants gave an overview of the different SDOH affecting patient populations:

The neighborhood around our practice has, you know, folks that have a lower socioeconomic standpoint, so that’d be one, lower education rate as a as another. So, food insecurity is another social determinant. We also look at social connectedness as another one, and finances as a fifth.

Participant 4

I would define social determinants of health as those factors that impact patients’ access to care, patients’ understanding of their care, and maybe the resources that are available to them. I think things outside of - yeah, those things that are sometimes kind of hard to quantify and it’s certainly hard to elicit.

Participant 3

Another participant focused in on health literacy as a salient problem among patients, further adding limited provider time reduces opportunities for health education:

You know, so a lot of our patients, you know, you can break it down to the sixth-grade level, and it’s still over their head. You know, it’s like, you know, the population when it comes to understanding, having what I call “health intelligence”, are not even at the second-grade level. So, social determinants of health, you know, education is a joke, our healthcare system doesn’t allow the health care providers the time they need to teach the patients and then of course, on the patient side, they just gave up. Now, that’s not all of them, that’s just most of them.

Participant 2

Participant 5 explained how using SDOH can help understand patient challenges and improve outcomes:

If I look at, just in general, my work in mental health, the patients are very often hindered from whatever they want, because of other things like finances, or they don’t have food. So, for example, I’ve served as a home health social worker, and I was only pulled into the cases where they weren’t getting better because of the social determinants of health. Obviously, if you’re not eating, your wound won’t get better, right? Obviously, if you’re about to be evicted and you’re taking care of your loved one who’s dying, you can’t take care of them, you’re working on the eviction.

Participant 5

The issue of access (whether it be constraints on time, transportation, and childcare) highlighted by participant 1 illustrates the importance of supportive health care services that attend to the entire family (parent caregivers) as well as patients.

I think the biggest social determinant and social justice problem that I see is with our patients – I mean, the parents of these patients – is their access, and their availability to be able to be with their babies at this very, very vulnerable time. So, making sure they have what they need and the equal opportunity to be able to access their child is a big problem that we see.

Participant 1

### Assessment and Documentation Practices

While 2 participants were familiar with International Classification of Diseases Z codes (Z55-65) specific to SDOH, 3 were not. Only 1 participant reported the use of these Z codes to actively document patient SDOH, indicating the codes were recommended by their EMR system to be added to the problem list and visit encounter. Three participants documented SDOH via provider notes or the patient education or care plan, and 2 participants indicated an automatic screener.

All participants agreed that assessing and documenting patient SDOH was very important and beneficial for improved health outcomes; however, most (n=4) noted SDOH are not systematically or consistently assessed. One participant stated asking whether SDOH is standard social work practice. Another participant mentioned that while sometimes more basic questions are asked, there is often no in-depth assessment, especially for outpatient care:

The focus is on the disease process and how to -- which medication to prescribe. I’ve seen it before, you know, I mean, they don’t really dig deeply these days. Now, if we’re talking about a medical student, or medical resident, or patients being admitted, man do they dig. But when you get out into a practice, like outpatient, stuff like that -- the social determinants really are boiled down to a few very minor things that are “yes” or “no” questions or basic answers when they fill out an application.

Participant 2

I think everybody, if we’re intervening with the patient, I think it is [our] ethical and moral responsibility to ensure that we’re addressing the needs of the entire patient, because it’s not just a medical need, or the daughter doesn’t just take care of the medical needs, or the nurse just doesn’t take care of nursing needs; I mean we take care of the whole patient in their family.... It doesn’t need to be this conversation of, “Oh, well we don’t talk about these things,” or “This is just the way that it is.” I mean, we’re all human beings and we all deserve the right to access equal access and equal opportunity.

Participant 1

When asked, “How often do you or does someone in your practice ask patients about social determinants of health?” no participants reported having a universal protocol to assess patient SDOH at each visit. All participants indicated they will either start to notice patterns or patients will specifically bring SDOH up over time, despite not actively screening for them. One participant stated they ask the patients questions about their lives in a conversational manner at each visit to glean information about SDOH, and 1 participant indicated an annual electronic screener all patients attending their practice receive. One participant said they ask at each initial appointment if the patients are affiliated with one of their grants requiring assessment of certain SDOH.

### Referrals to Other Providers and Community-Based Resources

When asked what is done when a need is identified, all 5 participants reported they refer patients to a variety of external providers and local community partners who provide health and social services such as new parent education, counseling and substance use services, and vocational rehabilitation. Specific national organizations participants mentioned included Ronald McDonald House, United Way, and First Steps, and 1 participant mentioned partnering with 2 community organizations through the NowPow platform [17]. One participant specified “attributed life” patients with certain insurance plans are eligible for referrals (official work orders) to the care coordination wing of the health care facility, bypassing the in-office social worker. One participant, a social worker, reported they actually provide the client with a list of suggestions for outside services, including legal or welfare benefits assistance, and this is documented in the patient’s case notes along with whether or not the patient followed up.

Three participants indicated they would make contact with an internal provider, such as a social worker, or to a provider who does semiregular drop-ins, such as a nutritionist, physical therapist, or podiatrist. Two participants reported directly addressing the need on-site at the time of the encounter:

We do have that very close-knit relationship [within our care team] where we can just go and talk to [our providers] face-to-face and be like, “Well, we need to get somebody to come address this, or we just need to, you know, have an intermediate interdisciplinary meeting, where we have everybody there at the same time.” Things like that. As far as transportation, we can get with social work to try to get like taxi vouchers or bus vouchers or whatnot.

Participant 1

So, there are some organizations that will help. It’s just a matter taking the time and making the phone call. I find if you give the patients the information and ask them to cold call, they don’t seem to. So, we kind of take the groundwork, and [if] they have a contact person, I think it seems to work a little bit easier. We’ll bring them in the office. I’ll bring them in my office, and we’ll make the call and see if we can’t get them.

Participant 3

### Barriers and Facilitators of SDOH Assessment and Documentation

Participants offered their perspectives on barriers to assessment and documentation of SDOH (see Multimedia Appendix 2 for participant quotes specifically focused on the theme of barriers and facilitators). When asked, “What makes it difficult to assess and/or document patient social needs?” the majority of participants referred to the system-level barrier of profitability, with the most frequent responses relating to insurance or billing demands and limits to time with patients. Two participants reflected on how revenue generation, for better or worse, is a driving force in modern clinical practices.

Although participants identified several limitations in relation to billing, there was acknowledgment insurance companies are attempting to be more holistic when it comes to patient assessments and to reimburse clinics more appropriately when they have patients with complicated health issues. In addition, currently, Z codes are not reimbursable, so there is little incentive for clinic personnel to take the time to enter them.

From the microlevel perspective, patient reluctance and a sense of stigma were mentioned most frequently. For example, patients may find SDOH questions intrusive and not the purview of a medical provider. However, all participants perceived patient-provider trust and rapport as a facilitator of SDOH assessment, noting it is important to be conversational with patients, to “normalize” the questions, and to build long-term relationships. The next most frequently perceived barrier was an inability to address the patient’s SDOH, whether due to a lack of provider knowledge as to how to help the patient when an issue was identified or a lack of available resources or services to which the patient could be referred.

### SDOH Assessment and Documentation Training Preferences

Participants identified continuing education credit-eligible brown bag sessions (informal meetings or trainings typically occurring over a lunch break), web-based courses, catered dinners, and destination conferences as their general preferences for training formats**.** Two participants stated they had training in assessment of SDOH during their nursing or social work education, while the other 3 expressed they have learned through experience. The 2 participants involved in training the health care workforce acknowledged this is still being done at the educational level and felt additional training targets, such as patient-provider simulations, could be incorporated into the curriculum.

We do something like this [with our students], like they get X amount of stipend, like with their paycheck, and then they have to go to all the community sources and make sure they can get groceries. But then I gotta pay the light bill, and this is like, they realize how quickly sometimes their money can go. Even incorporating that, now you’ve been hospitalized, and you could incorporate that into a lot of nursing education.

Participant 1

From a standpoint on how to ask the question, we do well. I guess part of my job is actually to help train the residents and medical students on how to ask the questions. And, so we do more of video feedback for the residents, and such.... But, for the attending faculty like myself, that is not necessarily something that they do for us per se. So, I’ve kind of more learned on my own, when it comes to that.

Participant 4

While only 1 participant reported having training on the use of Z codes from the “billing aspect,” the majority of participants did not feel training on SDOH assessment and documentation would be very beneficial without a system-wide push for a standardized protocol.

Four participants stated they would not benefit from training on how to ask patients about SDOH; 1 participant felt they would benefit from training to improve their knowledge of Z codes and more efficient documentation to save time.

Yeah, I guess I think about, you think about behavioral change, or you think about practice improvement or quality improvement, is that the more that you can do it a higher functioning level where it doesn’t rely on the individual person to change that actually makes it a more sustainable model. And so, giving a doctor education on the importance of health literacy, I think in the end is the least helpful long term. It needs to be something where it is with, like in the EMR, how the social determinants of health from the screener say, you know, “here’s some ICD-10 codes you should add to your bill,” and all you gotta do is click a few buttons. Because, I can scan it and agree, like “yep, yep, yep”, and then I can move on.

Participant 4

## Discussion

### Principal Findings

Overall, participants in our study conceptualized the SDOH of their patient populations in accordance with HP 2030 and could indicate how individuals in their care were affected by the 5 HP 2030 SDOH domains. Microlevel social needs identified by participants included material constraints such as time (to be with their child in the hospital), transportation, financial and food access barriers, and social disconnectedness. Identified SDOH at the macrolevel included poor education quality, with specific attention to a lack of health education that can contribute to limited knowledge of positive health behaviors and limited health literacy on the individual level. All participants mentioned the broader aspects of the SDOH domain of health care access and quality when describing the barriers to assessing and documenting SDOH during the clinical encounter (ie, time constraints and inconsistent documentation protocol).

### Organizational Challenges to Assessment and Documentation of SDOH

Participants realized the context of one’s life has a crucial role in the development and management of health conditions. All participants agreed assessing and documenting patient SDOH were very important and beneficial for improved health outcomes but most (n=4) noted it is not done consistently. Consistent with findings by Heidari et al [18], participants in our study mentioned several barriers regarding the assessment of SDOH and appropriate referrals, including a lack of universal screening protocol and little incentive to screen for SDOH or to use Z codes. Medical providers have very limited time with patients, so any additional information gathering can be difficult to accommodate consistently if not designed to seamlessly (efficiently) integrate within the health care encounter.

Despite the lack of formal methods of screening, all participants noted patient social needs are often revealed over time, whether through direct patient disclosure or indirect indicators of barriers to treatment adherence (eg, inability to read a prescription label, lack of transportation, and food insecurity). While 2 participants delivered annual or semiannual formal SDOH assessments to some or all of their patients, all participants suggested a more conversational approach often served to elicit depth of information on patient SDOH. However, even in instances where SDOH information is gathered informally, it can be difficult to act upon the information, since resources can be difficult to find or unavailable. This can lead to providers becoming frustrated since they feel they cannot help their patients and are thus wasting valuable visit time.

Z codes, while they present a way to quickly and consistently record SDOH information, are not billable to insurance so they may be supplanted by billable codes when numbers of codes are limited. Entering codes was also seen by participants as an inefficient use of time, especially in for-profit settings, since entering nonbillable codes takes time away from the entering of billable codes. Billing code disincentives have created unintended consequences for individual clinics to inquire about SDOH or to use Z codes that must be addressed at the system level. For example, validated screeners may provide a way to gather SDOH information but must be implemented as a system-level policy. Thus, especially in for-profit settings, the use of Z codes must be aligned with economic goals. Insurance companies can already support SDOH screening by using value-based models that incentivize longer-term patient outcomes. Making codes for SDOH billable would help further move medical services in the direction of more holistic care [19]. Efforts to consistently and adequately address unmet patient social needs may require state-level actions, such as those being implemented in North Carolina through the Healthy Opportunities Pilot initiative [20,21]. While this serves as a potential model, it is unclear how this type of collective intervention may be impacted by state Medicaid expansion decisions, legal, and budgetary constraints [22]. Additional strategies are needed to ensure all patients’ social needs are addressed, regardless of income or type and level of health care coverage.

### Community and Societal Challenges to Clinical Documentation of SDOH

Participants perceived patient reluctance and apprehension of stigma as interpersonal barriers to the assessment of SDOH. There is some emerging evidence related to the stigmatization of the SDOH. Rather than an acknowledgment of the important ways in which the life context impacts our health and well-being, participants in this study alluded to the SDOH negatively, demonstrating SDOH are perceived as deficits rather than basic health and human rights, such as access to adequate food, income, education, transportation, supportive social networks, housing, freedom from racism, and freedom from other discrimination. This is in opposition to the intended assessment and documentation of patient SDOH as important social “vital signs” impacting health outcomes [23]. Addressing the SDOH speaks to an understanding of the context of health and demonstrates clinician and organizational knowledge and insight into the factors influencing equitable conditions for optimal health and well-being. The stigmatization of the SDOH undercuts opportunities to positively impact individual and population health outcomes [24]. In addition, providers should be intentional in communication practices, using language that carries less stigma to underserved or historically marginalized community members and incorporating questions surrounding cultural, linguistic, and spiritual patient needs [25].

The next most common perceived barrier was an inability to address the patient’s SDOH. Our findings are consistent with those of Kostelanetz et al [26], who reported clinician-perceived barriers to SDOH screening among acute care patients including limited resources to address social needs, limited or no time or staff allotted for SDOH screening, and lack of training to address existing social care needs. There is growing consensus for health system and organizational accountability in support of SDOH assessment and documentation by ensuring adequate resources for screening education, patient support services, and referral to institution resources, community organizations, and public health agencies and to create stronger partnerships with community organizations for care that extends beyond the scope of medicine.

Participants suggested trust and rapport facilitate the assessment of patient social needs and felt long-term patient-provider relationships and standardization of SDOH assessment can reduce patient perceptions of provider judgment and reduce a sense of a power dynamic. This creates significant tension for clinicians who predominantly practice within a biomedical model of care and have limited educational exposure or incentive to adopt a social-ecological model of health care that includes attention to the SDOH. Given the importance of patient-provider trust and rapport, clinician practice of cultural humility is necessary for equitable patient care. Cultural humility entails provider openness, self-awareness, and humbleness [27], and “incorporates a lifelong commitment to self-evaluation and critique, to redressing the power imbalances in the physician-patient dynamic” [28]. Further, increased patient-provider contact, transparency, physician competency regarding patient social contexts, and genuine demonstrations of care and authenticity in communication can improve patient trust [29,30].

### Challenges With Referrals Following Assessment of SDOH

While systematically assessing and documenting patients’ SDOH are extremely important, ensuring there is a clear next step that involves assisting patients with social factors that impact health through referrals to community resources and organizations is vital for improved health outcomes [31]. Participants referred patients to both external providers and local community partners, documenting these in the EMR regardless of the need for a direct referral. Some participants, particularly those practicing in more rural areas, expressed frustration over the lack of local services to meet some of their patient needs. In addition, the concept of “social prescribing,” or recommending patients who engage in civic, art, recreational, or other activities to improve overall health and well-being [32], encourages sustainable, socially engaged outlets for patients. To alleviate provider burden for identifying and connecting with adequate, vetted, and accessible referral sources, there needs to be a concerted effort to connect to or build a referral infrastructure (ie, NowPow) to reduce future time burdens. This would require partnerships across health-serving sectors, including hospital systems, government agencies, and community agencies. For example, referrals to community adult literacy centers can provide patients with assistance in reading and understanding general and health-related information to improve their health literacy [33]. Another example is the development of medicolegal partnerships to aid patients in gaining access to or redressing issues impacting a variety of social needs including housing, material assistance, disability, or supplemental income supports [34]. Additionally, more hands-on referral approaches, where the patient is assisted in making the connection with the referral source and the referral source adequately assists the patient in navigating complex processes (ie, public benefit or housing applications), may eliminate barriers to “referral uptake” for better health outcomes [35].

### Limitations

This study has some limitations. First, we used purposive and convenience sampling to recruit participants. Second, the number of participants interviewed was quite small (N=5), limiting our ability to obtain findings generalizable to a specific health system, to the state of South Carolina, or to other US states. Third, participants had a range of years of experience within the health care field; however, some were newer to their current roles and thus had less experience within their current health care system. This poses a potential limitation for data source triangulation. Future research is warranted with additional providers to further explore assessment and documentation practices, barriers, and facilitators in additional settings in South Carolina. Examining provider and clinic practices by medical specialty and geographic location as well as proximity to referral resources will be important for supporting patients’ social needs following any assessment, documentation, or patient-provider discussions about SDOH. Despite these limitations, findings from our work will help providers and health care systems consider how to effectively integrate SDOH screening during patient-provider encounters, as findings offer important insights into providers’ perspectives and recommendations for SDOH assessment and the barriers and facilitators associated with it.

### Conclusions

SDOH have a clear and direct impact on individual and community health status. Clinical providers understand these ties; however, they struggle with the best approach to assess and document SDOH issues in clinical settings. Time constraints in clinic visits, perceived stigma of SDOH, and preference for a more conversational approach to the patient-provider discussion add complexities to data collection, which result in incomplete and inconsistent interactions across populations. Perhaps an institutional culture shift is required to ensure consistent screening and assessment of patients for unmet social needs and provision of appropriate resources, including connections to organizations in the community to assist with these needs. Unfortunately, the stigma surrounding SDOH issues, social isolation, and financial hardships continues to pervade society. Bringing these challenges into the open and talking to patients about them are necessary if the goal is to improve the overall health of patients.

## Appendix

Multimedia Appendix 1 Interview guide.

Multimedia Appendix 2 Barriers and facilitators of social determinants of health (SDOH) assessment and documentation.

## Abbreviations

EMR: electronic medical record
HP 2030: Healthy People 2030
SDOH: social determinants of health

## Data Availability

The data sets generated during and/or analyzed during this study are available from the corresponding author on reasonable request.
