# Supplementary material for: Assessment and Documentation of Social Determinants of Health Among Health Care Providers: Qualitative Study
Source: JMIR Form Res. 2023 Jul 3;7:e47461. doi: 10.2196/47461 (PMC10365596; doi:10.2196/47461)
Supplement: Multimedia Appendix 1 [file formative_v7i1e47461_app1.docx]

Thank you for your willingness to meet today. We are having these discussions with providers across the state to better understand your patients’ social needs and the use of social determinants of health ICD-10 Z codes. These discussions will help inform trainings and resources that will be developed to improve communication with patients.

Before we begin the discussion questions, I have a few questions about your clinical background, if that’s okay?

1. What is your current title and role?
2. How many years have you been in this role at this clinic?
3. How many years have you been working directly with patients in a clinical setting?

Interview:

1. How would you define “social determinants of health” as they pertain to your clinic patients?
2. How beneficial is it to assess and document patient social determinants for improved health outcomes?
   1. How often do you or does someone in your practice ask patients about social determinants of health? (***Interviewers: make sure we get at who is asking about SDoH)*
   2. In your opinion, do you think all practitioners/medical settings should screen for social determinants, or are there specific practitioner types that should focus on this. Why do you think so?
3. Are you familiar with ICD-10 SDoH Z codes? *If “yes”, move to question 3.1 below.

   If “no”, first share the following, then move to question 3.1 below:
    ICD-10-CM codes Z55-65 are designed to capture social determinants of health, which are referred to as
    “potential health hazards related to socioeconomic and psychosocial circumstances”.*

   3.1 When you assess patient social determinants of health, how do you document them and how often do
    you document them? (*Probes: EMR notes, Excel spreadsheet, Z codes; each visit? annually?*)
4. Once a need is identified, how do you help these patients?
   (*Probes: refer patients to other services, discuss how the impact presenting issue, etc.*)
   1. If you refer patients, what kind of services and service providers do you make referrals to? (
      *Probes: help with transportation, financial assistance, food security, mental health, etc.)*
   2. (If yes to referrals…) Do you partner with any community organizations for patient referrals?
   3. How do you document these referrals for social determinants of health?
5. What makes it difficult to assess and/or document patient social needs?
   (*Probes: time, knowledge of relevant Z codes, etc.)*
6. What types of training (if any) have you received on documenting patient social determinants of health and using ICD-10 Z codes?
7. Do you feel that you would benefit from training/resources to help you identify and address social determinants of health with your patients? (If “no”, why not?)

If “yes”:

- 1. What content or skills would you value most from training/resources related to social
     determinants?
  2. What type of format would you prefer for this?

Our final question for today is:

1. Is there anything else that you have encountered or that you think might be helpful to us that we didn’t ask about today?

*Thank you for taking the time to speak with me today. The information you shared is extremely helpful as we move forward to address health literacy and social determinants in South Carolina.*
