# Supplementary material for: Assessment and Documentation of Social Determinants of Health Among Health Care Providers: Qualitative Study
Source: JMIR Form Res. 2023 Jul 3;7:e47461. doi: 10.2196/47461 (PMC10365596; doi:10.2196/47461)
Supplement: Multimedia Appendix 2 [file formative_v7i1e47461_app2.docx]

| **Profitability** | | |
| --- | --- | --- |
| Issue | Barriers | Facilitators |
| Time constraints | - *So, our patients have really good [medical] outcomes here compared to other clinics I've been to because we have the luxury of time, and we're not fighting with insurance companies, and I don't have an MBA [administrative or finance person] telling me I have four minutes per patient. We have time.*   *I'm telling you, if you look at the surveys, what the physicians miss the most, when they used to be able to sit down and listen to their patients and have a personal relationship with them and not feel whether we're working on a treadmill…The insurance companies, [control] the purse strings for everything. That's why I don't do private practice anymore. I work at a free clinic. I don't have to deal with all that. But I have friends of mine who fight it every day. And it's just demoralizing. The whole system is broken. And who suffers? Patients?*  *So, I have a problem with bean counting. But the thing that matters is, you know, you can't measure quality outcomes. You can't, you have to have a personal relationship with the patients and their family members and see how their lives are improving, from their own mannerisms, how they talk to you, or they tell you their stories. And that's, that's how - I can’t put it on spreadsheets. We've become a nation of bean counters, and this is why medicine is failing. We're separated from the patients now. We’re all number generated now. We're numbers.*  *The [health care provider], they’re under the impression of pressure by the system; you’ve got to see patients so fast, in order to be profitable. And, if they’re not profitable, then [their] salary is a drag on the profitability of the company, which has shareholders and board members, and it’s become very corporate. And because of that, people get abbreviated health care.* [Participant 2]   - *In our practice we are not going to be seeing somebody just for food insecurity, or just for housing issues, we are going to glean that because we're taking care of their pregnancy. So, we're gonna be billing for the pregnancy…This is gonna sound ugly, I think we get $25 for completing the [SDoH] form. You know. I mean I think that's why the office has probably pushed for us to do that, because it generates revenue, you know. I’ve done this long enough to say that you know this is my when health care became about making, in making money we lost sight of why, we do why we're doing what we're doing. You know, we work, I work for a for-profit institution. I can't remember who owns us now, rural hospitals. We get sold all the time. So, they keep track of, you know the bottom line. But every time I see something like this, I start the, you know we need to do better. We need to do better.* [Participant 3] | - *We know that social determinants impact a person's utilization of health care and if they utilize health care in a cost-effective manner or not…From an insurance standpoint – from, you know a research standpoint – if you don't get it documented and sent to the insurance company, then, they don't realize sort of how complex your patients are. And one thing we're starting to see, maybe not so much in South Carolina, but across the country, is that they are starting to look at a complex view of your patients and social determinants starting to play into that. So, they'll actually reimburse you at a better rate, if you're able to demonstrate the complexity of the patients you're taking care of, right? So, that's the idea is that the insurance companies are starting to try to say, “Okay, we recognize that these kinds of patients are gonna take more time, more effort within the office. The doctor, you know honestly [can't do] it all. You have to bring in team members to help you out.* [Participant 4] |
| Non-reimbursable codes | - *One of the downsides is that, I think from a billing standpoint, you could actually only input 12 diagnoses into the chart. And, so once you've met that number, you have to prioritize which ones you put in with that particular bill.* [Participant 4] - *Z codes are the things insurance doesn’t pay for. So, why would I use them right? So, we have a lot of patients who have no insurance. We have grants, so the grant pays for it, whether they have Z code or not. The insurance won't pay for Z code so there's not… So, I get dinged all the time, once you put in as a code like, grief is a code that insurance won't pay for. And, yeah, grief isn't an illness, it's normal. So, I get that about insurance.* [Participant 5] | N/A^a^ |
| **Stigma** | | |
| Issue | Barriers | Facilitators |
| Patient assumption of provider judgment | - *I can't speak for everybody, but I think it's stigma that it is still segmented…You know, it's all of our jobs to be into these things [assessing SDoH] that sometimes, like [culturally], we're like, "Do we get involved in your business, or do we not get in your business?" And so, I think until, and then how is that perceived by others? Like, well, "How do you know? You don't know anything about me?" Or, "How can you Just assume that I don't have." So, I think it's on both sides.* [Participant 1] - *My concern is always, you know, sometimes, even when I ask the question, people assume that there is judgment, that because I'm asking the question, I've already made a judgment or an assumption about them, because I'm asking the question. But it always worries me that people are embarrassed or ashamed, or just not comfortable…And, a lot of times I’ll preface it with, “we ask this to every everybody”. You know, it's kind of like at that initial visit in your pregnancy, we screen everybody for sexually transmitted infections. Married single, young, old. We screen everybody. We drug screen everybody, we don't pick and choose who. So, a lot of times I'll couch it with, “These are questions we ask everybody”. You know, but I always worry that they're not going to feel comfortable sharing at that first visit.* [Participant 3] | - *I don't necessarily come out and blatantly say, you know, "What do you need? What are your…", but, I mean, in our population, [it’s like], "Well, you know, I haven't seen you in a couple of days, has everything been okay?". Or, we know there's other children at home, how are they doing? Or, you know, if you see mom and not dad, or dad, not mom? Or, if you see grandma and no parents, you kinda try to delve into it, and not be so blunt with like, “Why, haven't you been here, or you know, like, “Do you have transportation? Do you have money? Do you have this…” I mean, so, it's being very tactful and very, very purposeful in your conversations.* [Participant 1] - *I sit down, say what brings you here today, and then I look at their past medical history, and then I say, “What’s your life like? What's going on home?” And I read them, and I'm really good at reading people. Mostly kinda like, kind of “sit and spit”, “have you had good experiences with health care people in the past?” And, they open up. You know, so there's a way I can dig, you know. How am I different than every other doctor they’ve ever seen? So, I sit down, I actually shut my mouth. And listen.* [Participant 2] - *You know, I pride myself on being kind of very nonjudgmental, but very, just kind of easygoing and conversational, you know. Sometimes I feel like 90% of what I do is just sit and chat, and you know, kind of get comfortable with one another. It's good fun. Yeah, I mean these women are delightful.* [Participant 3] - *I think developing that long term relationship is the other piece that they would be able to be willing to say, “I didn't get that and that”, and you know that the patient feels heard in general and then you're not just sort of taking care of the current issue and trying to get out the door, that they you know that they can ask those follow-up questions, okay, so developing that relationship.* [Participant 4] |
| Perceptions of patient shame or embarrassment | - *Honestly patients are more willing to share sometimes with those different team members (social worker, health coach, care coordinator), than they are with the doctor. Sometimes they're a little bit more ashamed to share some of those things with the doctor…Getting used to being able to ask those questions in a way that doesn't come across like you’re – that the person would feel ashamed in sharing it and that they don't have to be embarrassed by it, is…it takes an art to do that as well.* [Participant 4] - *I think embarrassment makes it hard…I'm working with somebody right now that's super dependent. She has a lot of social determinants of health. So, her issue, she's embarrassed about it, very embarrassed. I've offered her some help, but she's also not the type to sort of take the ball and run with it, and so I can give her resources, but she wants me or somebody to do it. That's not my role, to do the job for her. So, that gets in the way, too.* [Participant 5] | - *Because, I mean, it's just like any patient or any person, if you make them feel uncomfortable, they're not gonna talk to you, and they're not gonna, you know, build that trust and that rapport that you really, really need…A lot of our babies are there for a long time. We do build that relationship with this team, and they feel more comfortable talking with us as, you know, we're not just the clinician anymore. We become more of a friend and a confidant to them.* [Participant 1] - *So, these people, they gotta learn who they can trust, you know; that you're not just going to throw them a prescription, back up a dump truck and throw pills at them. They’re human beings for crying out loud. You know, they just, they want you to be interested in them. And, that's where you get the trust, you know, if it's too sterile, and you're not looking at them, and listening to them, and you're just typing stuff with a scribe, you know, and it's all sterile. And, you know, forget about it. I’ve had physicians like that, like, you know, you just like you're already looking at the door handle.* [Participant 2] - *I think that's the beauty of pregnancy. My patients will see me for all but 2 of their visits. You know the docs' patients will see them all but 2 visits. We rotate our patients at least once with each other’s, so we've got a whole span of time where we can kinda warm up to each other.* [Participant 3] - *So, I treat a bunch of people with trauma. So, one of the first things I’m doing is, “You can trust me, I’m safe”. And, so it can take a while until they'll get to the place -- so I would say people with trauma, you know they might not be able to reveal it. And, you just have to figure that out.* [Participant 5] |
| **Patient SDoH referral resources** | | |
| Issue | Barriers | Facilitators |
| Knowledge and availability of resources | - *I think one of the challenges is knowing what to do with the response from the patient. You don't want to assess something that you don't have a resource to be able to offer, you know, if someone tells you they don't have food for the next day and all you can respond back is like, “Wow, that has to be rough”. That feels very empty. So, I think that's one of the hard things.* [Participant 4] - *I once had an instructor who said, “Be very careful what you take on in the office, as far as non-medical issues.” She said, “you need to be willing to finish whatever it is that you've started.” On the flip side of that is we don't have a great referral source, just anywhere to send these women…Sometimes I feel like we're out here in the, you know, country all by ourselves.*   *You come to rural South Carolina where there's not a public transportation system, where one of the churches here has lunch, I think 2 or 3 days a week. You know, where people live in homes without windows, and without electricity, and without running water. You know, stuff that I had never ever kind of considered until I moved south. You know, I applaud a lot of these women for doing as well as they do*. [Participant 3]   - *Frankly the problem is less that the doctors don't know what they're doing, or asking the right questions, or don't have the resource lists in front of them, it's that the resources aren't there. They don't exist. This is the state of South Carolina, and I should add to that it's the country the United States of America, where we don't care. Right, I’ve lived in several other countries where they do actually care, and these things do exist. So, that's part of the frustration providers have, is the resources.* [Participant 5] | - *You know, we have [name of organization] come here with their counselors, medical counselors, that talk to our patients, they have a big ole bus in the parking lot.* [Participant 2] - *So [health care system] has a platform called NowPow, which, essentially, they work with community partners to be on this platform. And so, a social determinant of health comes up via the screener that information for that organization is automatically brought into the after-visit summary or the education the patient gets at the end of the visit for resources to help them with those things that they might be struggling with.* [Participant 4] |

^a^N/A: not applicable.
